# Autophagy as a multi-scale architect of fungal development and pathogenicity: membrane dynamics, multilayer regulation, and cell wall integrity crosstalk

**DOI:** 10.3897/imafungus.17.196033

**Published:** 2026-07-09

**Authors:** Feixiang Wang, Jiarui Li, Huojuan Chen, Qiangwang Zheng, Tao Wei, Kun Feng, Bai-Xiong Chen

**Affiliations:** 1 School of Bioengineering, Zunyi Medical University, Jinwan Road No. 368, Zhuhai 519090, Guangdong, China Institute of Food Biotechnology & College of Food Science, South China Agricultural University Guangzhou China; 2 Institute of Food Biotechnology & College of Food Science, South China Agricultural University, Guangzhou 510642, Guangdong, China School of Bioengineering, Zunyi Medical University Zhuhai China

**Keywords:** Cell wall integrity, entomopathogenic fungi, epitranscriptomic regulation, host-pathogen interactions, fungal autophagy, morphogenesis

## Abstract

Autophagy is a conserved membrane-trafficking pathway traditionally viewed as a nonspecific nutrient recycling mechanism. However, recent advances across diverse fungal systems, from plant pathogens to human opportunistic fungi and entomopathogenic species, have revealed autophagy as a central regulatory hub that orchestrates fungal development, virulence, and host interaction at multiple biological scales. This review provides a comprehensive and critical synthesis of these emerging insights. At the nanoscale, the discussion explores how autophagosome biogenesis depends on the spatially precise delivery of PtdIns4P by oxysterol-binding proteins, the dual function of the TRAPPIII vesicle-tethering complex, and the retromer-mediated sorting of vacuolar proteases. At the organelle level, the interplay between selective autophagy (mitophagy, lipophagy, pexophagy) and a newly discovered layer of epitranscriptomic, transcriptional, and post-translational regulation, comprising m^5^C RNA methylation of core ATG transcripts, FOX transcription-factor-driven gene activation, and nuclear acetylation of Atg8, respectively, is examined. At the macroscale, the review highlights how autophagy-dependent cell death and ferroptosis cooperate to drive appressorium maturation in *Magnaporthe oryzae*, and presents direct biochemical evidence for crosstalk between the cell wall integrity MAPK cascade and the autophagy machinery, a paradigm that challenges the long-standing view of these pathways as parallel systems. Further discussion addresses how autophagy deficiency triggers Mincle-dependent host immunity in *Cryptococcus neoformans* and how entomopathogenic *Cordyceps militaris* co-opts autophagy for fruiting body morphogenesis. We emphasize that the direct biochemical evidence for several of these mechanisms, notably CWI–MAPK/Atg4 crosstalk and autophagy–ferroptosis coupling, currently derives largely from *Magnaporthe oryzae*, and we distinguish such established mechanisms from cross-species extrapolations throughout. Finally, Atg4 inhibitors are evaluated as a promising class of broad-spectrum antifungal agents, and key directions for future research, including spatiotemporal imaging, multi-omics validation, and translational antifungal strategies, are identified.

## Introduction

### The dual survival challenge of fungi and the global remodeling by autophagy mechanisms

Fungi have evolved remarkable adaptability across highly diverse ecological niches (Naranjo‐Ortiz and Gabaldón 2019). Whether it is the plant-infecting rice blast fungus (*Pyricularia oryzae*, syn. *Magnaporthe oryzae*), the Fusarium head blight pathogen (*Fusarium graminearum*) (Chen et al. 2025a), or the human pathogen *Cryptococcus neoformans* latent within macrophages (Gerstein et al. 2019), all of these fungi inhabit highly stressful niches. The same challenge extends to the cuticle-degrading *Metarhizium* (Gao et al. 2020), and to insect-pathogenic fungi that require complex life cycles to form macroscopic fruiting bodies (e.g., *Cordyceps militaris*): they must precisely coordinate internal material recycling with external cellular and tissue structural remodeling under extreme environmental stress.

Fungi inhabiting different niches exhibit distinct demands for morphological plasticity. Plant pathogenic fungi must rapidly transform from highly polarized tubular hyphae into massive, dome-shaped appressoria, generating immense physical turgor pressure to mechanically breach the host plant cuticle (Müller and Scheuring 2024). Human pathogenic fungi must maintain the integrity of their cell walls (Bloom et al. 2021) and capsules under the host’s oxidative stress and immune phagocytic pressure (Ruiz-Baca et al. 2025). For higher filamentous fungi with a fruiting body developmental stage, the transition from a diffuse vegetative hyphal network to a highly dense primordium, and ultimately to a mature fruiting body, demands spatial reallocation of massive biological macromolecules and extreme energy expenditure (Zhang et al. 2023).

In this grand and complex narrative of cellular reprogramming, autophagy plays an irreplaceable role. Autophagy itself is a membrane remodeling process involving dynamic interactions among multiple organelles. In recent years, the application of isotope pulse labeling combined with quantitative mass spectrometry (nPL-qMS) has enabled the precise, global tracking of fungal proteome turnover under nutrient fluctuations, confirming the synergistic role of bulk and selective autophagy in remodeling the entire fungal proteome (Telusma et al. 2025). Viewing autophagy merely as a metabolic pathway is no longer sufficient; we must integrate dimensions such as vesicle trafficking (Rayens et al. 2023), epigenetics, post-translational modifications (Lei and Klionsky 2023), multicellular developmental networks (Li and Du 2024), and host immune responses (Ding et al. 2021) to dissect how the autophagic machinery acts as a “ precise regulatory mechanism” to sculpt fungal cell fate and dictate development and virulence.

This review concentrates on pathogenic fungi, where the genetic, biochemical, and cell-biological evidence for autophagy is currently most complete. Findings from saprotrophic and symbiotic systems, including *Saccharomyces cerevisiae* (the foundational ATG-discovery model), *Komagataella phaffii*, *Aspergillus oryzae*, and *Serendipita indica*, are drawn upon where directly relevant to illustrate the conservation of the underlying machinery.

## Coordination of membrane lipid dynamics and intracellular trafficking networks: the basis of autophagosome biogenesis and maturation

The initiation of autophagy is a highly ordered membrane remodeling event (Liu et al. 2023). Its morphological manifestation in fungal cells depends not only on the assembly of core autophagy-related (Atg) proteins (the conserved set of more than 40 ATG-family members defined in **Overview of the core autophagy machinery**) but also deeply relies on the extensive intracellular lipid transport and vesicle trafficking networks (Hu and Reggiori 2023). Unlike the multiple ER-associated “omegasomes” (Nähse et al. 2024) broadly distributed in mammalian cells, autophagosome biogenesis in yeast and many filamentous fungi is typically highly concentrated at a specific subcellular compartment known as the Phagophore Assembly Site (PAS) (Kumari et al. 2025). Under fluorescence microscopy, the PAS appears as a highly dynamic, discrete punctate structure composed of the Atg1 kinase complex and the class III PI3-kinase complex (see **Overview of the core autophagy machinery** for their molecular composition) (Yasuda et al. 2026). When fungi face nutrient deprivation or cell wall stress in the host environment, the isolation membrane begins to elongate from the PAS (Shree et al. 2024). High-resolution electron tomography reveals that this elongation is not a simple membrane expansion but involves the generation of extremely complex geometric curvatures (Bieber et al. 2022). To form a double-membrane isolation membrane with highly curved edges, fungal cells must rapidly mobilize massive amounts of lipids from donor organelles such as the endoplasmic reticulum (ER) (Shi and Suzuki 2024). Under electron microscopy, “three-way junction” ultrastructures connecting the isolation membrane edges tightly with ER cisternae can be clearly observed (Gómez-Sánchez et al. 2025). Specific proteins (such as autophagy-related proteins containing BAR domains (Song et al. 2024)) aggregate at this site, physically intercalating to force the flat lipid bilayer to undergo severe curvature, ultimately forming a cup-shaped structure that closes into a mature autophagosome (Liu et al. 2023).

In this microscopic membrane remodeling process, the cytoskeleton system acts not merely as a passive support for fungal morphology (Hu and Reggiori 2022), but as an active participant in autophagosome formation and long-distance transport (Liu et al. 2023). Fungal mycelium is one of the most remarkable single-celled structures exhibiting polarized growth in nature; its apical extension and material transport heavily rely on the crisscrossing intracellular microtubule and actin cable networks. In the very early stages of autophagy, branched actin specifically polymerizes around the PAS to form a cage-like structure (Monastyrska et al. 2008; Hu and Reggiori 2022). This actin cage physically restricts the uncontrolled, random diffusion of the isolation membrane in the crowded cytoplasm and provides the mechanical counterforce necessary for directed membrane elongation (Capitanio et al. 2023). Once the autophagosome is closed, due to the high polarity of fungal cells, autophagosomes scattered throughout the hyphae must traverse distances of dozens of micrometers to be delivered to the hydrolytically active vacuole (equivalent to the mammalian lysosome) (Liang 2023). At this stage, specific motor proteins are recruited to the autophagosome surface, anchoring it to microtubule tracks for high-speed, centripetally polarized transport (Zwilling and Reggiori 2022). Under extreme stress conditions, fungi can also optimize autophagosome transport efficiency to the vacuole by modulating tubulin acetylation levels to enhance microtubule stability, ensuring highly efficient material recycling during the critical window of infection.

The assembly of the PAS and the elongation of the autophagosomal membrane require massive lipid influx (Yang et al. 2021; Yasuda et al. 2026). Recent studies have revealed, with unprecedented resolution, the core role of lipid molecules and transmembrane transport complexes in this process (Schütter et al. 2020; Dabrowski et al. 2023).

### Overview of the core autophagy machinery

Macroautophagy, the default meaning of “autophagy” throughout this review, is a conserved eukaryotic process in which cytoplasmic cargo is sequestered within a *de novo*-formed double-membrane phagophore, sealed into a mature autophagosome, and delivered to the vacuole (the fungal counterpart of the mammalian lysosome) for hydrolytic degradation (Nakatogawa 2020). Microautophagy involves direct invagination of the vacuolar membrane without an autophagosome intermediate, while chaperone-mediated autophagy is a HSC70/LAMP-2A–dependent pathway restricted to mammalian cells.

The molecular execution of macroautophagy depends on a conserved set of more than 40 autophagy-related (Atg) proteins, originally identified in landmark genetic screens in *Saccharomyces cerevisiae* by Ohsumi and colleagues (Tsukada and Ohsumi 1993), and subsequently shown to be largely conserved across fungi, plants, and animals (Nakatogawa 2020). Within this machinery, the Atg1/ULK1 kinase complex integrates upstream nutrient and stress signals to nucleate the phagophore at the phagophore assembly site (PAS); the class III PI3-kinase complex (Vps34, Vps15, Atg6/Beclin-1, and Atg14) generates the PI3P signal required for nucleation; the Atg9 transmembrane system delivers lipid material to the growing phagophore; and two ubiquitin-like conjugation cascades, the Atg12-Atg5-Atg16 system and the Atg8-phosphatidylethanolamine (Atg8-PE) lipidation system, drive phagophore expansion and closure. Atg7 functions as a critical bottleneck of the conjugation cascades, serving as the sole E1-like enzyme for both the Atg12–Atg5 and Atg8–PE systems.

Autophagy operates in non-selective and selective modes. Under acute nutrient deprivation, non-selective (bulk) autophagy randomly engulfs cytoplasmic contents to liberate amino acids and lipids for cellular reuse. Selective autophagy, in contrast, uses cargo receptors that link specific substrates to the Atg8/LC3-decorated phagophore through Atg8/LC3-interacting motifs (AIMs/LIRs); the major subtypes relevant to this review are mitophagy, pexophagy, lipophagy, ER-phagy, nucleophagy, and ferritinophagy (Vargas et al. 2023). A brief note on the relative weight of yeast/fungal versus mammalian evidence in the field is warranted. Although autophagy is more extensively studied in mammalian systems today, the molecular identification of the core machinery was carried out almost entirely in *S. cerevisiae*, and most named ATG genes therefore retain their yeast-derived nomenclature even in mammalian and filamentous fungal contexts. Fungal and mammalian autophagy systems also differ in three notable respects. Autophagosomes nucleate at a single, vacuole-proximal PAS in yeast and most filamentous fungi but at multiple ER-associated omegasomes in mammalian cells. The single yeast *atg8* has expanded into at least six mammalian paralogs (LC3A/B/C, GABARAP, GABARAPL1/2), each with distinct cargo-receptor preferences. Finally, autophagosomes fuse with small, motile lysosomes in mammalian cells but with the typically large, central fungal vacuole, a topology that places much greater spatial demand on cytoskeleton-dependent autophagosome transport in filamentous fungi.

### Precise spatial rearrangement of lipid transport proteins and PtdIns4P

**Figure 1.**
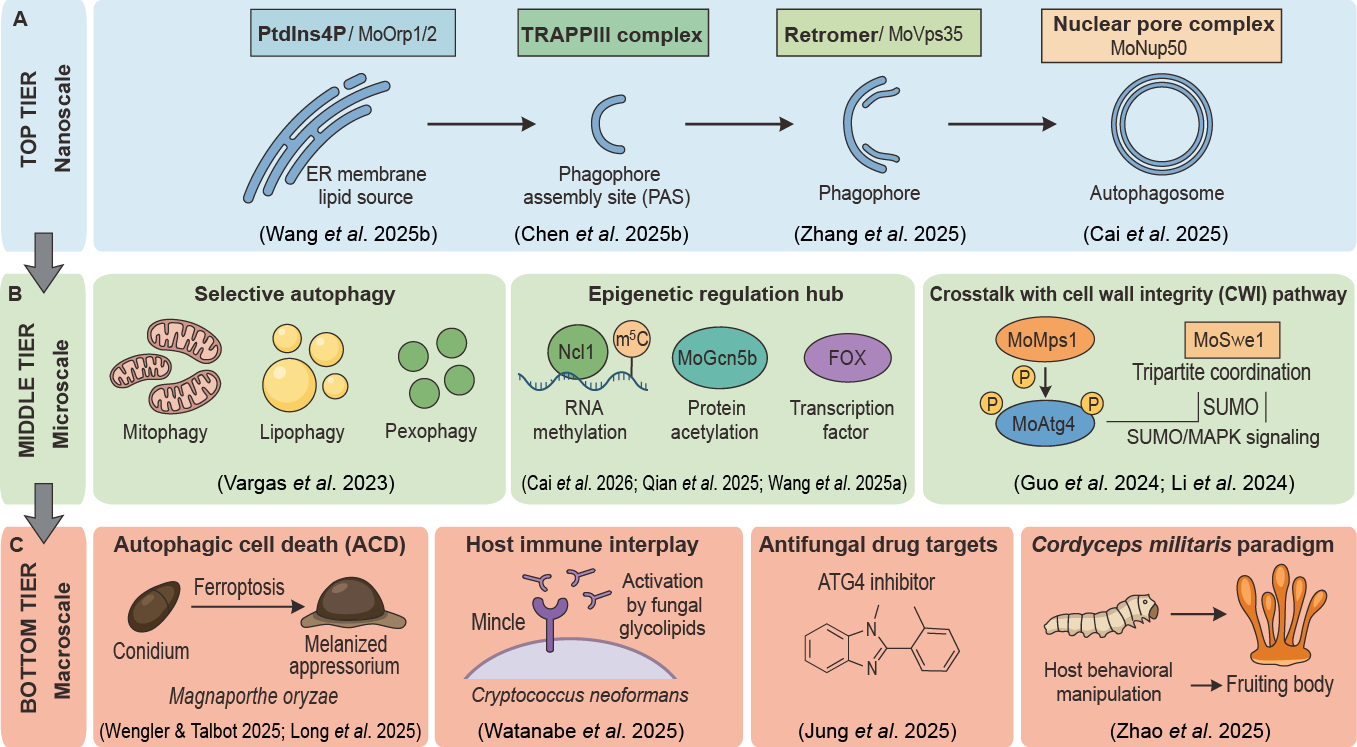
Overview of the multi-scale autophagy regulatory framework in fungal morphological plasticity. The diagram is organized into three spatial tiers. **A** Top tier (Nanoscale): autophagosome membrane biogenesis from ER-derived lipids through the phagophore assembly site (PAS) to mature autophagosomes, highlighting the roles of PtdIns4P/MoOrp1/2, TRAPPIII complex, Retromer/MoVps35, and nuclear pore complex MoNup50. **B** Middle tier (Microscale): selective autophagy types (mitophagy, lipophagy, pexophagy), the multilayer regulation hub (m^5^C RNA methylation, protein acetylation, FOX transcription factors), and direct crosstalk with the cell wall integrity (CWI) pathway (MoMps1–MoAtg4 phosphorylation, MoSwe1 tripartite coordination, SUMO/MAPK signaling). **C** Bottom tier (Macroscale): functional outputs including autophagic cell death (ACD) with ferroptosis in *M. oryzae*, Mincle-dependent host immune activation in *C. neoformans*, ATG4-targeting antifungal strategies, and the *Cordyceps militaris* host manipulation paradigm.

Phosphatidylinositol-4-phosphate (PtdIns4P) plays a critical role in autophagosome biogenesis (Zhang et al. 2021) and membrane fusion (Fig. 1A) (Levin et al. 2017). In *M. oryzae*, the oxysterol-binding protein-related proteins MoOrp1 and MoOrp2 have been identified as specific transport carriers for PtdIns4P (Wang et al. 2025b). Biochemical analyses indicate that these two proteins can physically interact with the core autophagy protein MoAtg8 and are recruited to the PAS (Shi et al. 2024). At this location, MoOrps are responsible for precisely transporting and enriching PtdIns4P onto the forming autophagosomal membrane (Laczkó-Dobos et al. 2024). This MoOrp-dependent spatial rearrangement of PtdIns4P is not only a prerequisite for the formation of mature autophagosomes but also an absolute requirement for the successful subsequent fusion of autophagosomes with the vacuolar membrane (Kwon et al. 2025). The double gene knockout mutant (Δ*Moorp1-2*) suffers from a complete blockade of autophagic flux due to the inability to efficiently accumulate PtdIns4P on autophagosomes and vacuolar membranes, ultimately causing the pathogen to lose the ability to generate appressorial turgor pressure and penetrate host cells (Wang et al. 2025b).

### Cross-boundary regulation by the vesicle trafficking hub TRAPPIII complex and the nuclear pore complex

In this microscopic membrane remodeling and transport process, the vesicle tethering complex TRAPPIII (Transport Protein Particle III) plays a bridging role in cross-boundary regulation (Fig. 1A) (Zhao et al. 2017). In *Fusarium graminearum*, the TRAPPIII complex - containing specific subunits FgTrs85, TRAPPC11, TRAPPC12, and TRAPPC13 - not only localizes to the PAS but also directly interacts with the sole transmembrane autophagy protein FgAtg9 via FgTrs85 and TRAPPC13, promoting the anterograde transport of FgAtg9 to ensure smooth autophagosome biogenesis (Chen et al. 2025b). Strikingly, this complex simultaneously functions as a Guanine Nucleotide Exchange Factor (GEF) to activate the small GTPase FgRab1, thereby orchestrating bidirectional intracellular vesicle transport from the ER-to-Golgi and from endosomes-to-Golgi (Chen et al. 2025b). This dual functionality of TRAPPIII perfectly coordinates the exceptionally high demands for autophagy and secretory pathways during sexual reproduction (perithecium formation) and the infection stage (Chen et al. 2025b).

Furthermore, recently discovered Nuclear Pore Complex (NPC)-associated proteins are also deeply embedded in the fungal autophagy regulatory network (Sumner and Brickner 2022). In *M. oryzae*, the nuclear basket protein MoNup50 resides at the nuclear envelope but can specifically interact, both *in vivo* and *in vitro*, with the core E1-like enzyme MoAtg7 (a critical bottleneck of the Atg8-PE lipidation cascade, as detailed in **Overview of the core autophagy machinery**) (Shi et al. 2024). MoNup50 not only plays a crucial role in maintaining appressorial cell wall integrity and mediating osmotic stress- and cell wall integrity-responsive MAPK signaling through MoOsm1 (the high-osmolarity glycerol/HOG pathway MAPK homolog) and MoMps1 (the terminal MAPK homolog in the cell wall integrity pathway), but also functions as a negative regulator of autophagy (Lu et al. 2023; Cai et al. 2025). Its deletion leads to abnormal accumulation of the lipidated MoAtg8-PE form and hyperactive autophagy, which ultimately significantly attenuates the pathogen’s infectivity due to disrupted glycogen and lipid droplet metabolism (Cai et al. 2025).

### Spatial sorting of vacuolar proteases by the retromer complex

To ensure the highly efficient execution of autophagic degradation, the precise delivery of the vacuolar hydrolytic enzyme system is equally vital. The Retromer complex plays an indispensable role in safely transporting hydrolytic enzymes into the fungal vacuolar lumen (Zhang et al. 2025). In *M. oryzae*, the core subunit of the Retromer complex, MoVps35, is responsible for recognizing and binding the serine protease MoPrb1 on the endosomal membrane, directing its transport into the vacuole (Fig. 1A) (Zhang et al. 2025). Once inside the vacuole, MoPrb1 further recruits and assists in the correct spatial localization of the vacuolar aspartic protease MoPep4 (the fungal homolog of *Saccharomyces cerevisiae* Pep4/proteinase A, which is responsible for activating downstream vacuolar hydrolases upon proteolytic maturation). Biochemical and genetic analyses have unveiled a clear division of labor: MoPrb1 specifically mediates the degradation of macroautophagy cargoes related to infection structure development, and its mutation leads to appressorium development failure; whereas MoPep4 independently governs microautophagy, such as the autophagic degradation of ubiquitinated peroxisomes (Pexophagy), though it is dispensable for fungal virulence. The discovery of this pathway perfectly illustrates how the vesicle sorting machinery governs the precise delivery of distinct proteases, thereby selectively controlling the operation of macroautophagy and microautophagy pathways (as defined in **Overview of the core autophagy machinery**) (Zhang et al. 2025).

## Adaptive organelle remodeling and multi-dimensional modifications: from morphological interplay to the precise convergence of post-transcriptional and post-translational regulation

We use these terms in their strict sense: m^5^C methylation of ATG mRNAs is epitranscriptomic, FOX-mediated control is transcriptional, and acetylation, phosphorylation and SUMOylation of Atg proteins are post-translational; classical chromatin-level regulation of fungal autophagy remains comparatively unexplored. Under environmental stress, fungal organelles themselves undergo morphological evolution, and the massive intracellular molecular machineries exist in a highly dynamic state of remodeling (Fig. 1B). When fungi confront host immune attacks or environmental stressors, organelles do not passively await as “cargo” for autophagic degradation; there is a profound intrinsic interplay between their morphological evolution and selective autophagy (the receptor-mediated, cargo-specific subtype of autophagy introduced in **Overview of the core autophagy machinery**, in which dedicated receptors such as Atg32 or MoAti1 link specific cargoes to the Atg8-decorated phagophore via AIM/LIR motifs). Behind this remodeling lies an exquisitely orchestrated network of epitranscriptomic, transcriptional, and post-translational regulation.

### Selective autophagy of organelle networks, morphological interplay, and spatiotemporal sanctuary

Taking the methylotrophic yeast *Komagataella phaffii* as an example, global analysis using nPL-qMS revealed that upon shifting carbon and nitrogen sources, up to 90% of the pre-existing fungal proteome is remodeled (Telusma et al. 2025). This massive reconstructive process is primarily driven by Atg9-dependent bulk autophagy, but is concurrently accompanied by highly specific organelle-selective autophagy (Peng et al. 2021). Specifically, peroxisomes are rapidly degraded via Atg30- and Atg11-dependent pexophagy, whereas mitochondria are progressively cleared at a slower rate via Atg32-dependent mitophagy (Barone et al. 2023). At the molecular level, these two selective autophagy pathways are independent and non-competitive (Miller et al. 2020; Telusma et al. 2025). Even more intriguingly, under steady-state growth conditions, the fungal mother cell can spatiotemporally “shield” specific maternal peroxisomes from degradation via the plasma membrane-anchoring protein Inp1, showcasing remarkable flexibility and survival strategies during organelle remodeling (Telusma et al. 2025).

Such flexibility is also manifested in the morphological interplay of mitochondria themselves. As the cell’s energy factory and redox center, mitochondrial dynamics, fusion and fission, directly dictate the fate of mitochondria in the autophagic flux. In *M. oryzae*, the dynamin-related GTPase MoDnm1 (the fungal ortholog of mammalian DRP1 and yeast Dnm1) has been demonstrated to mediate both peroxisomal and mitochondrial fission by assembling, via the WD-40 adaptor MoMdv1, into a complex with the outer-mitochondrial-membrane anchor MoFis1; this complex is required not only for asexual conidiation and full virulence but also for the execution of pexophagy and mitophagy under nitrogen starvation (Zhong et al. 2016). Genetic disruption of Modnm1, Mofis1, or Momdv1 impairs mitochondrial fragmentation, blocks mitophagic clearance of foot-cell mitochondria during conidiation, and severely attenuates appressorium-mediated host penetration. These findings establish that, in pathogenic fungi, regulated mitochondrial fission via the MoDnm1–MoMdv1–MoFis1 complex is a developmentally programmed prerequisite for mitophagy-coupled morphogenesis, rather than a passive byproduct of mitochondrial damage. The size scaling between fragmented mitochondria and the engulfing isolation membrane is also of mechanistic importance: when mitochondria remain as elongated tubular networks, their dimensions exceed the typical encapsulation capacity of autophagosomes (~1 μm in diameter), potentially limiting their accessibility to canonical autophagic degradation (Sakurai et al. 2022). Upon MoDnm1-mediated asymmetric fission triggered by oxidative damage or membrane potential depolarization within host cells, the resulting small, spherical daughter mitochondria become compatible with the geometric constraints of the phagophore, enabling efficient mitophagic engulfment. This size-matching logic, fission as a prerequisite for selective engulfment, appears to be broadly conserved across fungi and underlies the coordinated mitophagy programs that fuel infection-structure maturation and invasive growth (Zhong et al. 2016; Shi et al. 2024). Besides mitochondria, the autophagic degradation of lipid droplets (LDs) (Lipophagy) similarly exhibits astonishing membrane morphological ingenuity, which is especially critical in the life cycles of insect-pathogenic fungi (Rahman et al. 2021). For entomopathogenic fungi such as *Metarhizium robertsii* and *Beauveria bassiana*, dormant conidia store large amounts of triacylglycerol-rich lipid droplets (Wang et al. 2022). Upon attachment to the insect cuticle and initiation of germination, the fungus mobilizes lipid droplets (LDs), generating carbon sources and acetyl-CoA that support the development of penetration structures (Lei et al. 2022). However, LD diameters are often much larger than typical double-membrane autophagosomes, posing the spatial and geometric paradox of “being too large to be engulfed at once.” Morphological observations suggest that large lipid droplets (LDs) can be degraded in a piecemeal manner, where the isolation membrane locally assembles and invaginates along the LD surface, allowing partial sequestration of LD material during lipophagy (Jung et al. 2023). Second, through a microlipophagy pathway, the vacuolar membrane locally invaginates and sequesters lipid droplets (LDs) or portions thereof into the vacuolar lumen, where they are degraded by vacuolar lipases (Rahman et al. 2021). Deletion of *atg8 homologs* (e.g., *Mratg8* or *Bbatg8*) disrupts autophagosome formation and is associated with defects in lipid droplet (LD) utilization, leading to impaired conidial germination and reduced host infectivity (Lee et al. 2022). In opportunistic human pathogens and plant pathogenic fungi, autophagy’s regulation of specific metabolites also dictates virulence. In *Colletotrichum gloeosporioides*, the core autophagy gene *Cgatg16* not only regulates vegetative hyphal growth and appressorium differentiation but also directly participates in the metabolic remodeling of melanin synthesis via the TOR and cAMP signaling pathways. Its absence significantly impairs the pathogen’s ability to penetrate hosts like rubber trees (Cheng et al. 2025).

### m^5^C RNA methylation: the epitranscriptomic “gatekeeper” of autophagy transcripts

**Figure 2.**
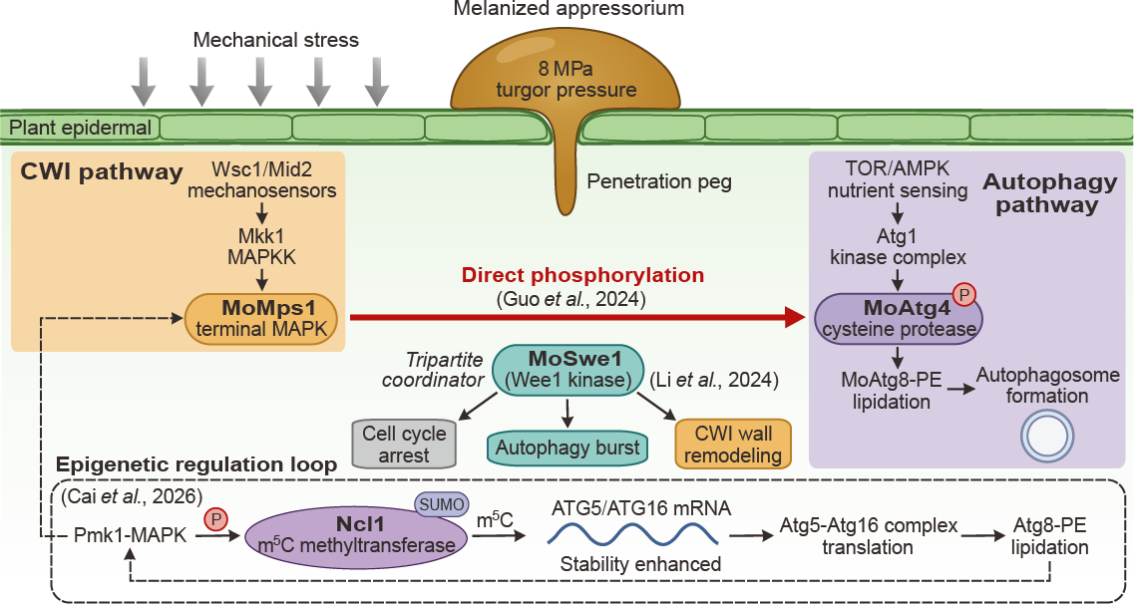
Molecular crosstalk between cell wall integrity (CWI) signaling and autophagy machinery during appressorium development in *Magnaporthe oryzae*. The melanized appressorium generates ~8 MPa turgor pressure against the plant epidermal surface. The CWI pathway terminal MAPK MoMps1 directly phosphorylates the autophagy core protease MoAtg4 (red arrow), independently of TOR signaling. The Wee1-family kinase MoSwe1 acts as a tripartite coordinator simultaneously targeting cell cycle arrest, autophagy burst, and CWI wall remodeling. The dashed box depicts the epitranscriptomic regulation loop in which Pmk1-MAPK phosphorylation and SUMOylation stabilize the m^5^C methyltransferase Ncl1, which enhances *atg5/atg16* mRNA stability to sustain Atg8-PE lipidation.

The complex physiological process of autophagy is governed not merely by biochemical substrates and morphological dynamics but is under strict surveillance by epitranscriptomic and post-translational regulation. A recent study introduced RNA methylation (m^5^C) into the autophagy regulatory landscape of plant pathogenic fungi for the first time. In *M. oryzae*, the m^5^C RNA methyltransferase Ncl1 acts as a critical “epitranscriptomic gatekeeper” determining the successful execution of autophagy. Ncl1 precisely adds m^5^C modifications to specific mRNA loci of key autophagy genes *atg5* and *atg16* (e.g., *C960*/*C1498* in *atg5* and the *3’ UTR* of *atg16*). This significantly enhances the half-life and stability of these transcripts, safeguarding the robust translation of the Atg5-Atg16 complex, which is a prerequisite for driving Atg8 lipidation and autophagosome assembly. Even more sophisticatedly, the stability of the Ncl1 protein itself is strictly controlled by dual post-translational modifications (Cai et al. 2026). On one hand, upstream kinases of the Pmk1-MAPK signaling pathway directly phosphorylate Ncl1 at the S491, T492, and T579 sites; on the other hand, Ncl1 must undergo Smt3-dependent SUMOylation (Fig. 2) (Liu et al. 2025). This complex cascade of “kinase phosphorylation/SUMOylation, methyltransferase stabilization, RNA stability enhancement – autophagy activation” establishes a perfect closed loop from the sensing of external host signals to internal post-transcriptional regulation.

### The transcription factor network and the acetylation switch of core proteins

At the transcriptional level, FOX (Forkhead box) family transcription factors build a direct bridge between fungal development and autophagy (Wang et al. 2025a). In *Sclerotinia sclerotiorum*, the causal agent of devastating stem rot, the transcription factor SsFoxE2 directly binds and activates the promoters of a battery of core autophagy genes, including *atg5*, *atg12*, and *atg13*. Upon starvation or developmental stress, the AMPK homolog SsSnf1 phosphorylates SsFoxE2, which is associated with increased transcriptional activity. This modification also promotes its interaction with SsTctp1 and contributes to the stabilization of SsFoxE2 by reducing ubiquitin-mediated proteasomal degradation (Mizuno and Irie 2021). The successful execution of this autophagic transcriptional activation program is an absolute prerequisite for *S. sclerotiorum* to overcome nutrient limitations and initiate early primordium development of the apothecium (fruiting body) (Zhu et al. 2025). Concurrently, post-translational modifications of core ATG proteins within the nucleus constitute critical regulatory nodes. In *M. oryzae*, the histone acetyltransferase MoGcn5b mediates the acetylation of MoAtg8 in the nucleus, and the dynamics of this modification directly determine the nucleocytoplasmic shuttling of MoAtg8 and the overall autophagic flux. Astonishingly, MoSec13, which is involved in vesicle trafficking, localizes not only to the ER and vesicles but also enters the nucleus. Utilizing specific amino acid residues (such as Ser111 and Gly134) in its WD40 domain, it interacts with MoAtg8 to prevent hyperacetylation by sterically hindering MoGcn5b binding. This acts as a negative fine-tuner for autophagic flux and lipid homeostasis (Qian et al. 2025). Together, these findings reveal that fungal autophagy is far from a simple biochemical reaction; it is a multi-dimensional network integrating organelle morphological interplay, epitranscriptomic and post-translational regulation, and cascaded post-translational modifications.

## Macroscopic morphogenesis and immune interplay: from cell death to immune evasion

Shifting the perspective from the molecular and organellar levels to macroscopic tissue and host interaction dimensions, the autophagic state of a fungus directly dictates whether the pathogen can successfully penetrate host physical barriers, and whether it evades or triggers the host’s innate immune system (Fig. 1B).

### The synergy between Autophagic Cell Death (ACD) and ferroptosis

In plant pathogenic fungi, one of the most disruptive discoveries in autophagy research is its capacity to drive highly programmed Autophagic Cell Death (ACD), which serves as a prerequisite for constructing specialized infection structures (e.g., appressoria) (Fig. 1C). When the conidium of *M. oryzae* germinates to form an appressorium on the plant surface, it must accumulate an enormous internal turgor pressure up to 8.0 MPa (Wengler and Talbot 2025). The source of this immense physical force relies not only on the synthesis of osmolytes like glycerol and the dense deposition of melanin in the cell wall but also strictly depends on the systematic collapse of its maternal structure: the three-celled conidium. As the appressorium matures on the plant surface, the cells within the posterior conidium initiate a massive, spatiotemporally strictly controlled massive autophagy induction (Eseola et al. 2021). Under transmission electron microscopy, the initially cytoplasm-rich conidium undergoes massive cytoplasmic vacuolization, accompanied by the frantic accumulation of double-membrane autophagosomes. Organelles, soluble proteins, and even nuclei within the spore are systematically engulfed and transported to the vacuole for degradation (Hashimoto et al. 2024). Crucially, these degraded macromolecules are not excreted as metabolic waste; instead, they are converted into foundational precursors continuously transported across septa into the developing appressorium. The moment the appressorium matures and prepares to penetrate the host, the maternal conidial cells completely collapse, marking a strict occurrence of Autophagic Cell Death (Long et al. 2025). This ACD holds profound physiological significance. It fundamentally differs from pathological necrosis associated with membrane rupture (Table 1). Moreover, mutations in core autophagy genes (like *Moatg8*) directly prevent the spore cells from dying, thereby completely blocking the macroscopic transfer of materials into the appressorium and rendering the mutant strain nonpathogenic and incapable of penetrating the host plant. Recent studies further reveal that accompanying this classical autophagic degradation (Wang et al. 2025b), a lipid peroxidation crisis triggered by autophagy-dependent iron release, known as ferroptosis, simultaneously unfolds in the conidial cells (Liu et al. 2022). Cargo-dependent selective autophagy degrades damaged mitochondria, releasing large amounts of reactive iron, which in turn leads to the lethal accumulation of lipid peroxides on the plasma membrane. This controlled ferroptosis works synergistically with autophagy, together determining the irreversibility of maternal cell clearance and material transfer, which is ultimately governed by upstream TOR, cAMP/PKA, and Pmk1-MAPK core signaling networks (Wengler and Talbot 2025). This developmental programmed cell death proves that in pathogenic fungi, death is not the end of life, but a highly programmed macroscopic sculpting process designated to “make way and supply energy” for novel morphological structures.

**Table 1.** Comparative morphological and biochemical features of major cell death types.

**Feature**	**Apoptosis**	**Necrosis / Necroptosis**	**Autophagic cell death (ACD)**	**Autosis**	**Methuosis**	**Ferroptosis**	**Developmental ferroptosis (fungal)**	**References**
***Morphological features***								
**Cell volume**	Marked shrinkage	Swelling (oncosis)	Maintained or slightly reduced	Early spreading, late swelling	Extreme swelling (vacuole accumulation)	Normal or slightly reduced	Progressive vacuolization of conidia	(Park et al. 2023; Lotfi and Rassouli 2024; Ji et al. 2025)
**Plasma membrane integrity**	Long-term intact, blebbing	Early rupture, content leakage	Intact until very late stage	Intact until late stage	Eventually ruptured	Lipid peroxidation-driven rupture	Intact until complete maternal cell collapse	(Park et al. 2023; Lotfi and Rassouli 2024; Ji et al. 2025)
**Nucleus**	Pyknosis, karyorrhexis, chromatin margination	Karyolysis, irregular chromatin clumps	No obvious condensation, nucleus relatively intact	Perinuclear space balloon-like swelling	Relatively normal until lysis	Relatively normal	Nucleus systematically degraded via nucleophagy	(Hashimoto et al. 2024)
**Cytoplasmic markers**	Dense organelles → apoptotic bodies	Mitochondrial swelling → lysis	Abundant double-membrane autophagic vacuoles	ER expansion, fragmentation, disappearance; abundant autophagosomes	Abundant single-membrane macropinocytic vacuoles (LC3–)	Mitochondrial cristae loss, increased membrane density	Massive double-membrane autophagosome accumulation; extensive vacuolization	(Long et al. 2025)
***Biochemical and signaling mechanisms***								
**Core biochemical mechanism**	Caspase cascade activation	RIPK1/RIPK3/MLKL pathway	ATG gene-dependent	Na^+^/K^+^-ATPase-dependent	Ras/Rac pathway hyperactivation	GPX4 inactivation; ferritinophagy releases free iron	Autophagy-dependent iron release → lipid peroxidation; ferroportin 1 autophagic regulation	(Liu et al. 2022; Long et al. 2025)
**Upstream signaling**	TNF/Fas; Cyt-c release	TNF/TLR signaling	TOR inactivation; AMPK activation	Beclin-1-dependent	Ras mutation; Rac1	Iron overload; Cys deprivation	TOR/cAMP-PKA/Pmk1-MAPK core network	(Wengler and Talbot 2025)
**Autophagy involvement**	Indirect (clearance of apoptotic bodies)	Indirect / inhibitory	Core executor (ATG essential)	Excessive autophagy as direct cause	Non-canonical (LC3– vacuoles)	Selective autophagy (ferritinophagy/mitophagy) as driver	Macroautophagy + selective autophagy synergize; ferroptosis feedback loop	(Long et al. 2025)
***Fungal pathogenicity relevance***								
**Role in fungi**	Host apoptosis post-appressorium maturation	Host tissue necrosis during infection	ACD drives penetration peg formation in *M. oryzae* (core mechanism)	Not yet reported in fungi	Not yet reported in fungi	Mammalian ferroptosis model	Irreversible conidial clearance in *M. oryzae*; unidirectional material transfer to appressorium	(Eseola et al. 2021; Long et al. 2025; Wengler and Talbot 2025)

### Molecular integration across pathways: precise crosstalk between autophagy machinery and Cell Wall Integrity (CWI) signaling

During the aforementioned critical window of drastic macroscopic morphological transition, the rate of internal material mobilization (autophagy) must synchronize exquisitely with the rate of external cell wall remodeling. This inherently necessitates cross-pathway molecular integration. For a long time, the molecular biology community considered the autophagy pathway (primarily responsible for sensing internal nutritional status) and the Cell Wall Integrity (CWI) pathway (primarily responsible for sensing mechanical stress and coordinating external cell wall remodeling) as two parallel, independently operating signaling cascades. However, breakthrough advances in recent years have uncovered a new paradigm of mechanosensory network regulation over autophagy (Müller and Scheuring 2024), with definitive direct biochemical evidence once again stemming from deep dissection of appressorium development in *M. oryzae* (Guo et al. 2024). The fungal CWI pathway is generally activated by a series of mechanosensors (e.g., the Wsc/Mid2 family) and transduced downstream to a core MAPK kinase cascade (such as the typical Mkk1-Mps1 module) (Shree et al. 2024). Recent biochemical and proteomic evidence startlingly revealed that at the critical juncture when the *M. oryzae* appressorium matures and faces powerful mechanical counterforces from the plant surface, the terminal MAP kinase of the CWI pathway, MoMps1, directly and specifically phosphorylates the cysteine protease MoAtg4, a core component of the autophagy machinery (Li et al. 2024). MoAtg4 acts as a “molecular scissor” in autophagosome formation, precisely exposing the C-terminal glycine residue of the ubiquitin-like protein Atg8 before its lipidation with phosphatidylethanolamine (PE)(Alam et al. 2024). Studies confirm that this direct phosphorylation modification mediated by MoMps1 can dynamically and precisely amplify or attenuate the autophagic flux within fungal cells independently of the classical TOR (target of rapamycin) nutrient-sensing pathway (Fig. 2) (Guo et al. 2024). This unprecedented kinase-substrate interaction establishes a direct biochemical bridge between “external structural stress sensing” and “internal autophagic membrane dynamic remodeling” at the molecular level, allowing the fungus to dynamically match its intracellular material recycling rate with the real-time physical pressure exerted during host penetration. More elegantly, during the morphological transition phase of infection structure formation, fungi have evolved even higher-order integration hubs alongside CWI-MAPK phosphorylation. In *M. oryzae*, the Wee1-family cell cycle regulatory kinase MoSwe1 has been proven to act as a crucial tripartite coordinator (Li et al. 2024). When the appressorium needs to breach the plant epidermis and extend a highly narrowed penetration peg, MoSwe1 simultaneously phosphorylates key targets in the autophagic machinery and key enzymes in the cell wall synthesis pathway, thereby acting as a molecular “synchronization switch” (Fig. 2). On one hand, it enforces cell cycle arrest to centralize biomass resources; on the other hand, it activates autophagy for massive mobilization of internal nutrient reserves, while spatiotemporally pinpointing local cell wall remodeling and degradation at the base of the appressorium (Li et al. 2024). This molecular tripartite integration of “cell cycle arrest - autophagy burst - CWI remodeling” vividly illustrates how fungi seamlessly bridge internal metabolic destruction with external structural construction at a macroscopic level. It is noteworthy that, although direct biochemical evidence for phosphorylation targets is currently concentrated in *M. oryzae*, genetic deletions of core autophagy genes have been widely observed to result in extreme hypersensitivity to cell wall-targeting drugs like Congo Red and echinocandins in human pathogens such as *Cryptococcus neoformans* (Ding et al. 2018; Roberto et al. 2020) and *Aspergillus fumigatus* (Roberto et al. 2020) (i.e., the cell wall stress phenotype caused by autophagy deficiency). This cross-phenotype, repeatedly manifesting across various evolutionary clades, strongly implies that the deep molecular binding between the autophagy machinery and cell wall stress-response networks is highly likely a core survival strategy retained throughout the long evolution of fungi (Table 2).

**Table 2.** Comprehensive summary of the multi-layered fungal autophagy regulatory network and antifungal translational strategies.

**Regulatory level / Type**	**Species**	**Key molecule(s)**	**Target / Substrate**	**Molecular mechanism**	**Functional effect & pathogenicity**	**References**
***I. Direct autophagy–CWI pathway crosstalk***						
**MAPK phosphorylation**	*M. oryzae*	MoMps1 (CWI MAPK)	MoAtg4	Terminal CWI kinase directly phosphorylates core autophagy protease MoAtg4, dynamically regulating autophagic flux independently of TOR	Bridges mechanosensory stress and autophagy; deficiency abolishes appressorial turgor	(Guo et al. 2024)
**Cell cycle–autophagy–CWI tripartite coordination**	*M. oryzae*	MoSwe1 (Wee1 kinase)	Autophagy components + cell wall synthases	Simultaneously phosphorylates autophagy machinery and cell wall synthases; synchronizes “cell cycle arrest–autophagy burst–CWI remodeling”	Molecular synchronization switch for penetration peg formation	(Li et al. 2024)
**Nuclear pore–autophagy negative regulation**	*M. oryzae*	MoNup50 (nucleoporin)	MoAtg7 / MoAtg8-PE	NPC protein interacts with MoAtg7 to negatively regulate autophagy; maintains CWI and osmotic stress MAPK signaling	Deletion → autophagy hyperactivation → glycogen/LD metabolic disorder → attenuated virulence	(Cai et al. 2025)
**SUMOylation–MAPK crosstalk**	*M. oryzae*	Pmk1 + SUMO pathway	Pmk1 phosphorylation; Ncl1 stabilization	SUMOylation regulates Pmk1 phosphorylation; Pmk1-MAPK stabilizes Ncl1 methyltransferase	Infection structure development; links CWI to epitranscriptomic regulation	(Liu et al. 2025; Cai et al. 2026)
**Genetic evidence (human pathogens)**	*C. neoformans*	Atg4/Atg8; ATG genes	Cell wall stress phenotype	ATG gene deletion → extreme sensitivity to Congo Red/echinocandins; reduced virulence	Conserved autophagy–CWI coupling across evolutionary clades	Roberto et al. 2020; Ding et al. 2018
**Mincle immune activation**	*C. neoformans*	EGCrP2/Sgl1	GFP-Atg8 trafficking	Sgl1 deletion → autophagic dysfunction → EG/AEG accumulation → EV release → Mincle activation → TNF-α/MIP-2 storm	Lethal at 37 °C; early pathogen clearance from host lungs	(Watanabe et al. 2025)
***II. Epitranscriptomic and post-translational modification regulation***						
**m** ^5^ **C RNA methylation**	*M. oryzae*	Ncl1 (m^5^C MTase)	*atg5/atg16* mRNA	Adds m^5^C at *atg5* C960/C1498 and *atg16* 3’UTR → enhances mRNA stability → ensures Atg5–Atg16 translation	Drives Atg8 lipidation and autophagosome assembly; essential for appressorial autophagy	(Cai et al. 2026)
**Dual modification of Ncl1**	*M. oryzae*	Pmk1-MAPK + Smt3	Ncl1 protein	Pmk1 phosphorylates Ncl1 (S491/T492/T579) + Smt3 SUMOylation → closed loop: signal → enzyme stability → RNA stability → autophagy	Multi-layered safeguard of epigenetic enzyme stability	(Liu et al. 2025; Cai et al. 2026)
**Nuclear Atg8 acetylation**	*M. oryzae*	MoGcn5b (HAT)	MoAtg8	Nuclear acetylation of MoAtg8 → regulates nucleocytoplasmic shuttling → determines autophagic flux	Acetylation promotes Atg8 nuclear export to activate autophagy	(Qian et al. 2025)
**Acetylation negative regulation**	*M. oryzae*	MoSec13 (WD40)	MoAtg8	WD40 domain (Ser111/Gly134) interacts with MoAtg8 → steric hindrance prevents MoGcn5b → prevents hyperacetylation	Fine-tuning of autophagic flux and lipid homeostasis	(Qian et al. 2025)
**Atg3/Atg9 acetylation**	*M. oryzae*	MoHat1 (HAT)	MoAtg3/MoAtg9	Histone acetyltransferase acetylates core autophagy proteins	Functional appressorium formation and pathogenicity	(Yin et al. 2019)
**FOX transcription factor**	*S. sclerotiorum*	SsSnf1 → SsFoxE2	Atg5/12/13 promoters	AMPK phosphorylates SsFoxE2 → enhances transcriptional activity → reduces ubiquitin-mediated degradation → activates Atg expression	Initiates apothecium primordium development	(Zhu et al. 2025)
***III. Selective autophagy types and functions***						
**Mitophagy**	*M. oryzae*	MoAti1→MoAtg8; Atg32	Damaged mitochondria	Damaged mitochondria asymmetrically fission → small spheres → isolation membrane engulfment; reactive iron triggers ferroptosis feedback	Clearance of depolarized mitochondria; developmental ferroptosis core	(Shi et al. 2024; Long et al. 2025)
**Lipophagy**	*B. bassiana*; *C. militaris*	*atg8 homolog*s	Lipid droplets	Isolation membrane piecemeal engulfment along LD surface; or vacuolar micro-lipophagy; releases carbon and acetyl-CoA	Conidial germination; Cordyceps fruiting body energy core	(Rahman et al. 2021; Lee et al. 2022)
**Pexophagy**	*K. phaffii*; *M. oryzae*	Atg30/Atg11; MoPep4	Peroxisomes	Ubiquitination → selective degradation; MoPep4 independently regulates microautophagy	Rapid degradation upon carbon switch; dispensable for virulence	(Telusma et al. 2025; Zhang et al. 2025)
**Nucleophagy**	*M. oryzae*; *A. oryzae*	ATG proteins	Nucleus / nuclear components	Nucleus engulfed by autophagosomes → vacuolar degradation	Systematic nuclear clearance during appressorium maturation	(Hashimoto et al. 2024)
**Ferroportin / iron-exporter autophagy**	*M. oryzae*	Ferroportin 1	Iron storage/transport proteins	Selective degradation of iron transporter → free iron release → lipid peroxidation	Induces developmental ferroptosis; regulates appressorium maturation	(Liu et al. 2022; Long et al. 2025)
***IV. Membrane trafficking hubs and autophagy crosstalk***						
**TRAPPIII dual function**	*F. graminearum*	FgTrs85/TRAPPC13	FgAtg9 anterograde transport	TRAPPIII interacts with FgAtg9 for autophagy; simultaneously acts as GEF for FgRab1 ER→Golgi secretion	Dual defects in sexual reproduction and infection	(Chen et al. 2025b)
**Retromer protease sorting**	*M. oryzae*	MoVps35 (Retromer)	MoPrb1 → MoPep4	Retromer recognizes MoPrb1 → vacuolar delivery → MoPrb1 recruits MoPep4; division of labor for macro-/micro-autophagy	MoPrb1Δ → appressorium failure; MoPep4Δ → virulence unaffected	(Zhang et al. 2025)
**PtdIns4P lipid signaling**	*M. oryzae*	MoOrp1/MoOrp2	Autophagosome/vacuolar PtdIns4P	MoOrps interact with MoAtg8 → transport PtdIns4P to autophagosomal membrane → drive autophagosome–vacuole fusion	ΔMoorp1-2 completely blocks autophagic flux → loss of penetration	(Wang et al. 2025b)
***V. Antifungal targets and translational strategies***						
**Atg4 inhibitors**	*C. neoformans*; *A. fumigatus*; *C. auris*; *M. oryzae*	Ebselen and derivatives	Atg4 catalytic Cys	Se forms reversible covalent bond with Atg4 catalytic Cys → blocks Atg8 maturation/lipidation → shuts down flux	Broad-spectrum fungicidal; BRET platform established	(Jung et al. 2025)
**CWI + autophagy dual-target**	*Broadly applicable*	Echinocandins + autophagy blockers	β-1,3-glucan synthase + autophagy cascade	Echinocandins inhibit cell wall synthesis + autophagy blockers sever material compensation → dismantles plasticity	Novel combination to overcome monotherapy resistance	(Ding et al. 2018; Roberto et al. 2020; Guo et al. 2024)
**m** ^5^ **C pathway intervention**	*M. oryzae*	Targeting Ncl1 or upstream	Ncl1/Pmk1-MAPK/SUMO	Disrupts Ncl1 stability → ATG mRNA degradation → autophagy transcriptome collapse	Epitranscriptomic antifungal strategy	(Cai et al. 2026)
**Mincle immune exploitation**	*C. neoformans*	Autophagy inhibitor adjuvant	EG/AEG metabolism	Autophagy inhibition → abnormal AEG release → enhanced Mincle-dependent innate immunity	Autophagy inhibitor-assisted immune clearance	(Watanabe et al. 2025)
**Cordyceps fruiting body model**	*C. militaris*	CmTreH1 (HGT)	Host trehalose → sugar crash	HGT trehalase → blood sugar crash → behavioral manipulation → “fatty pupa” → lipophagy/glycophagy → fruiting body	“Host manipulation → autophagy → fruiting body” paradigm	(Wang et al. 2025c; Zhao et al. 2025)

### Lipid metabolism disorder and mincle-dependent host immune activation

Precisely because autophagy is deeply tied to fungal cell wall and plasma membrane integrity, it serves as a decisive weight in the host-pathogen immune interplay in human fungal pathogens. In *Cryptococcus neoformans*, which causes fatal meningitis, the vacuole-localized steryl-β-glucosidase EGCrP2/Sgl1 is responsible for degrading ergosteryl-β-glucosides (EG) in the vacuole (Fig. 1C). Deletion of this gene leads to fatal autophagic dysfunction (manifested as severely blocked GFP-Atg8 trafficking to the vacuole) under host body temperature (37 °C) and nutrient deprivation, triggering massive fungal cell death. Even more alarmingly, autophagy defects lead to the abnormal intracellular accumulation of EG and its acylated derivatives (AEGs) in *C. neoformans*, which are then massively released into the extracellular environment via fungal extracellular vesicles (EVs). As highly potent glycolipid ligands, these released fungal AEGs are precisely recognized by the C-type lectin receptor Mincle on the surfaces of host dendritic cells and macrophages. By binding the fatty acyl chains of AEGs into specific hydrophobic lipid-binding pockets on the receptor, Mincle is robustly activated, unleashing a severe host inflammatory cytokine storm (e.g., massive production of TNF-α and MIP-2), completely clearing the pathogen from the host lungs during the very early stages of infection (Watanabe et al. 2025). This classic paradigm vividly illustrates how an internal fungal autophagic metabolic disorder directly translates into a high-intensity “danger signal” recognized by the host immune system. Similarly, in mutualistic interactions between symbiotic fungi (such as *Serendipita indica*) and *Arabidopsis thaliana*, the host’s autophagy system also functions as a critical “gatekeeper”. Host autophagy is locally activated by purine metabolites (dAdo) produced by fungal-secreted effector proteins, restricting fungal over-colonization and alleviating host cell necrosis caused by immune and metabolic stress, thereby ingeniously sustaining a win-win symbiotic homeostasis (Khan 2025).

### Novel broad-spectrum antifungal drugs targeting core ATG proteins

Given the absolute core position of autophagy in fungal survival, morphological remodeling, and pathogenicity, targeting autophagy-related proteins has emerged as one of the most promising strategies for developing next-generation antifungal agents. Current clinical and agricultural antifungal drugs (polyenes, azoles, echinocandins) are facing increasingly severe resistance crises (Hu et al. 2026). Focusing on the maturation and lipidation process of the core autophagy protein Atg8, the latest high-throughput drug screening has revealed that Ebselen and its derivatives can act as potent autophagy inhibitors (Jung et al. 2025). They form reversible covalent bonds between their selenium atom and the cysteine residue in the catalytic center of the Atg4 cysteine protease, directly and specifically inhibiting Atg4 catalytic activity. Both *in vitro* and *in vivo* experiments verify that such Atg4 inhibitors not only block plant pathogen infection but also broadly suppress the autophagic processes of various lethal human pathogenic fungi, including *C. neoformans*, *Aspergillus fumigatus*, and multi-drug-resistant *Candidozyma auris*, exhibiting exceptionally strong fungicidal activity (Fig. 1C) (Jung et al. 2025). Such autophagy-targeting small molecules, discovered utilizing Bioluminescence Resonance Energy Transfer (BRET) screening platforms, offer a highly promising novel avenue for overcoming the global challenge of antifungal drug resistance.

## The pinnacle of morphogenesis and behavioral manipulation: the pivotal role of autophagy in fruiting body development of entomopathogenic fungi

**Figure 3.**
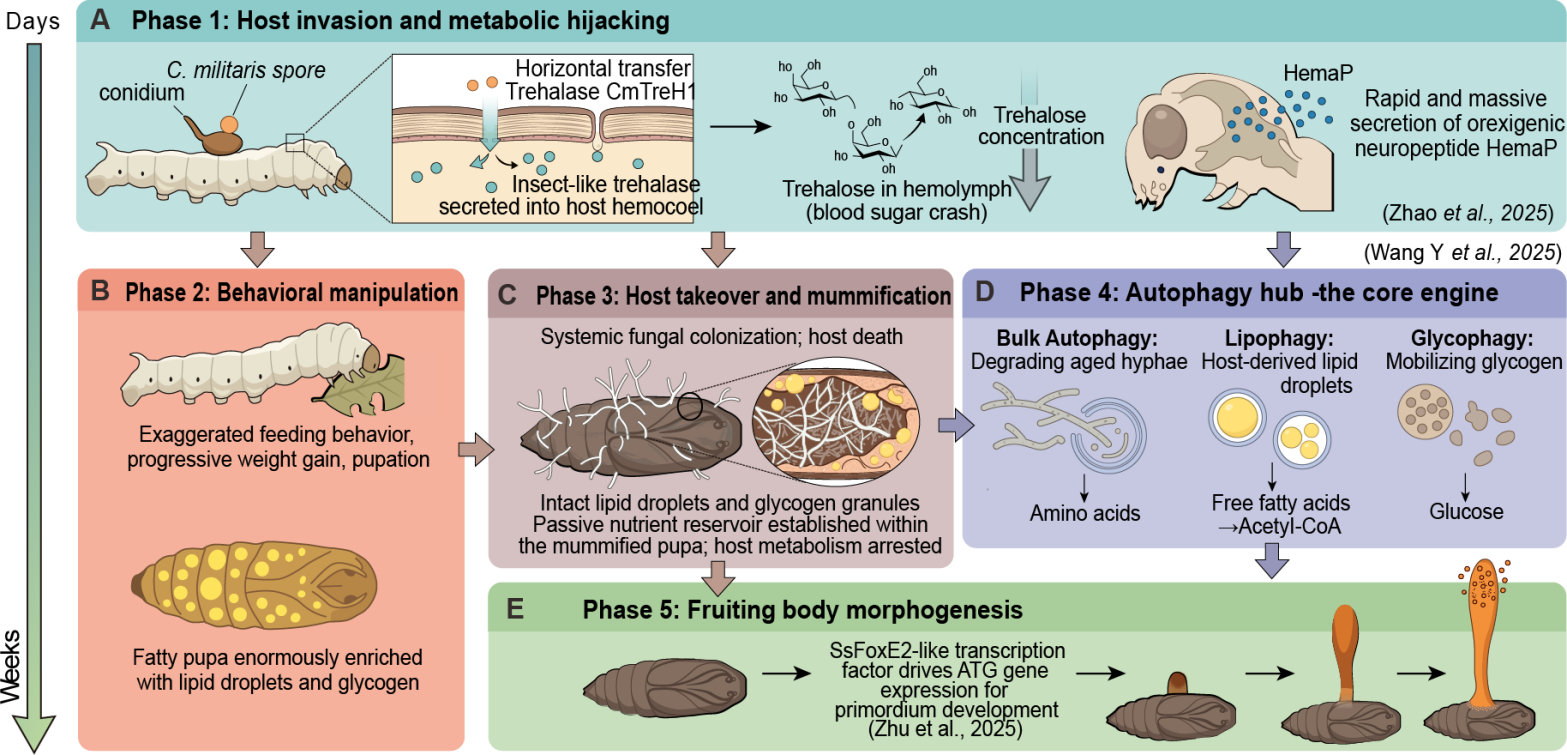
The *Cordyceps militaris* paradigm: from host behavioral manipulation to autophagy-driven fruiting body morphogenesis. The figure depicts five sequential phases (top to bottom) on a “days-to-weeks” timeline (left arrow). **A** Phase 1 (Host invasion and metabolic hijacking): fungal spores secrete the horizontally transferred insect-like trehalase CmTreH1 into the host hemocoel, catastrophically depleting hemolymph trehalose and triggering the massive secretion of the orexigenic neuropeptide HemaP in the host brain (Zhao et al. 2025). **B** Phase 2 (Behavioral manipulation): HemaP-driven hyperphagia leads to progressive caterpillar weight gain and pupation into a “fatty pupa” enormously enriched in lipid droplets and glycogen (Zhao et al. 2025). **C** Phase 3 (Host takeover and mummification): the fungus completes systemic colonization of the pupa, the host dies, and the mummified pupa becomes a passive nutrient reservoir filled with intact lipid droplets and glycogen for downstream fungal development (Lou et al. 2020). **D** Phase 4 (Autophagy hub, the core engine): three parallel autophagic programs cooperate to remobilize the stored nutrients, bulk autophagy degrades aged hyphal content (liberating amino acids), lipophagy dismantles host-derived lipid droplets (liberating free fatty acids and acetyl-CoA), and glycophagy mobilizes glycogen (liberating glucose) (Wang Y et al. 2025c). **E** Phase 5 (Fruiting body morphogenesis): the massive energy and carbon flux liberated in Phase 4 feeds the autophagy-fueled three-dimensional morphogenesis of the *Cordyceps* fruiting body, proceeding sequentially from primordium initiation through stroma elongation to the mature, perithecium-bearing club-shaped fruiting body. A *Sclerotinia sclerotiorum*-like FOX-family transcription factor program (SsFoxE2 analog) is proposed to drive ATG gene transcription during the primordium initiation phase (Zhu et al. 2025).

In the study of autophagy and fungal developmental biology, the single-celled appressorium of plant pathogenic fungi is frequently used as a primary model. However, higher entomopathogenic fungi represented by *Cordyceps militaris*, due to their extremely complex developmental axis spanning three sequential macroscopic stages: the vegetative hyphal network (the filamentous mycelial growth phase), the primordium (a dense, compact aggregation of differentiated hyphae that marks the initiation of sexual/reproductive development), and the mature fruiting body (the macroscopic, spore-bearing reproductive structure that emerges from the primordium and ultimately produces ascospores) provide an even more exquisite model system for understanding the role of autophagy in extreme macroscopic tissue remodeling (Fig. 3) (Lou et al. 2020; Zhang et al. 2023).

### Host behavioral manipulation: the “Extended Phenotype” creating a massive nutrient reservoir

Before entomopathogenic fungi can form fruiting bodies, they must hijack and stockpile massive nutritional resources from the host. Recent groundbreaking discoveries show that when *Cordyceps militaris* infects silkworm larvae and other insects, it does not rapidly kill the host (Ono et al. 2022). Instead, it exhibits an astonishing “behavioral manipulation” or “extended phenotype”. Through horizontal gene transfer (HGT) during evolution, *C. militaris* acquired an insect-like trehalase (CmTreH1) (Fig. 3A). During infection, this enzyme is abundantly secreted into the host hemocoel, catastrophically degrading and depleting the primary blood sugar (trehalose). This “blood sugar crash” physiologically mimics extreme starvation, robustly inducing the massive transcription and secretion of an orexigenic peptide, a neuropeptide that stimulates appetite and feeding behavior (e.g., HemaP in lepidopteran insects), in the host insect’s brain. Driven by this neuropeptide, the infected caterpillar develops an uncontrollable hyperphagia, leading to a significant increase in body weight and ultimately forming a “fatty pupa” highly enriched in lipids and glycogen upon pupation (Fig. 3B) (Zhao et al. 2025).

### The intervention of the autophagic machinery: from mummified pupa biomass to fruiting body morphogenesis

The parasitic strategy of *C. militaris*, manipulating the host to eat frantically to accumulate fat and glycogen, is fundamentally aimed at stockpiling colossal nutrient reserves for the later breach of the insect cuticle and the growth of massive fruiting bodies. After the host pupates and is completely taken over and killed by the fungus (Fig. 3C), the massive lipid droplets and glycogen stored in the fungal dormant structures inevitably require highly robust catabolic machineries for remobilization. At this juncture, autophagy (specifically Lipophagy and Glycophagy) becomes the absolute core hub connecting the “host nutrient reservoir” with “macroscopic fruiting body development” (Fig. 3D). The fungus must degrade aged hyphal contents via bulk autophagy, and extensively dismantle host-derived lipid droplets via selective autophagy, releasing massive amounts of amino acids, free fatty acids, and acetyl-CoA. This exceptionally high energy flux “feeds back” into the development of new primordia, sustaining the weeks-long development of the perithecium and ascospores. Although the direct functional characterization of core autophagy genes (e.g., *Cmatg8*, *Cmatg16*) in *C. militaris* has only just begun (Wang et al. 2025c), referencing the mechanism by which the transcription factor SsFoxE2 drives *atg* gene expression to initiate fruiting body primordium development in *S. sclerotiorum* (Fig. 3E) (Zhu et al. 2025), we can deduce that the autophagy system acts as the physiological engine driving this complex multicellular, three-dimensional morphogenesis in higher filamentous fungi.

## Conclusion and future perspectives

In summary, the role of autophagy in fungal cell biology and pathogenesis has far transcended the simplistic metaphors of an “intracellular recycling bin” or a “mere stress response” established in traditional paradigms. Instead, it represents a coordinated morphological remodeling, meticulously orchestrated by post-transcriptional regulators (such as m^5^C modifications), relayed by transcription factors and post-translational modifications, and executed synchronously by the cytoskeleton, vesicle trafficking systems, and membrane lipid macromolecules. From the bottom up, autophagy threads through the nanoscale generation of membrane curvatures, the microscale evasion and capture within organelle networks, up to the macroscale sculpting of infection structures and multicellular development. Furthermore, the autophagic machinery not only engages in direct phosphorylation-mediated crosstalk with external mechanosensory networks such as Cell Wall Integrity (CWI) but also acts as the decisive weight in the life-and-death struggle between pathogens and host immune systems (e.g., Mincle receptors). This proves that fungi possess a unified, highly efficient molecular messenger network integrating internal and external environmental morphological stresses. Looking ahead, breakthroughs in this field are urgently required across the following dimensions:

### Spatiotemporal tracking of autophagic dynamics and high-resolution imaging

Although transmission electron microscopy provides highly valuable static ultrastructural cross-sections, future studies must incorporate Correlative Light and Electron Microscopy (CLEM) and more sensitive, multi-channel live-cell fluorescent probes (e.g., specific fluorescent markers for the CWI pathway, endogenous lipid probes, and real-time confocal observation of GFP-ATG8). This will facilitate the dynamic, continuous spatiotemporal tracking of autophagosome bursts and their transient spatial interplay with the cytoskeleton and cell wall synthetic vesicles within *in vivo* infection models or complex three-dimensional developmental processes, deciphering the dynamic flux of macromolecular degradation.

### Epigenetic control and multi-omics cross-validation

Drawing upon the epigenetic modification mechanisms discovered in pathogenic fungi, future research should focus on exploring whether there are specific epigenetic modifications in higher filamentous fungi that trigger the instantaneous, massive derepression of autophagy-related gene clusters, acting as irreversible switches for morphogenesis - when switching from “vegetative growth” to “reproductive development,” or when coping with extreme host environments. Concurrently, phosphoproteomic and multi-omics interactome studies must cross species boundaries and be validated across a broader range of agricultural pathogens and human clinical fungi of significant public health concern. This will ascertain whether the cross-integration mechanisms between autophagy and stress pathways constitute a universal rule of life for filamentous fungi or represent specialized evolutionary adaptations to specific host niches.

### Targeted antifungal intervention and translational applications

From the practical standpoint of translational medicine and crop protection, the autophagic macromolecular network (e.g., targeting core components like Atg4) has emerged as an exceptional target for the development of next-generation antifungal therapeutics. If it can be further confirmed that cell wall repair or virulence in clinically lethal fungi heavily relies on the material compensation and signaling support provided by autophagy, then, in the future, combination therapy strategies utilizing “cell wall synthesis inhibitors combined with autophagic cascade blockers” may become highly promising, novel biological defense regimens capable of thoroughly dismantling fungal morphological plasticity and overcoming the challenges of monotherapy resistance.

## Additional information

### Artificial Intelligence (AI) use

The authors accept full responsibility for the content of the manuscript, including the disclosure of any use of AI.

Regarding the use of AI in the preparation of this manuscript, the authors declare the following: Gemini

Used for: Language, style and writing

During the preparation of this manuscript, the authors used Gemini (Google) solely to improve the English language, grammar, phrasing, and overall readability of the original draft. No AI tools were used to generate the scientific content, core arguments, conceptual framework, or references.

### Adherence to national and international regulations

All the fungal strains used in this study have been legally obtained, respecting the Convention on Biological Diversity (Rio Convention).

### Author ORCIDs

Q. Zheng https://orcid.org/0000-0001-8640-4513

T. Wei https://orcid.org/0000-0003-3816-8127

K. Feng https://orcid.org/0000-0002-2828-8232

B.-X. Chen https://orcid.org/0000-0003-0934-083X

### Data availability

All of the data that support the findings of this study are available in the main text.
